# Impact of improved dead time correction on the quantification accuracy of a dedicated BrainPET scanner

**DOI:** 10.1371/journal.pone.0296357

**Published:** 2024-04-05

**Authors:** Ahlam Said Mohamad Issa, Jürgen Scheins, Lutz Tellmann, Cláudia Régio Brambilla, Philipp Lohmann, Elena Rota-Kops, Hans Herzog, Irene Neuner, N. Jon Shah, Christoph Lerche

**Affiliations:** 1 Institute of Neuroscience and Medicine 4, INM-4, Forschungszentrum Jülich, Jülich, Germany; 2 JARA, BRAIN, Translational Medicine, Aachen, Germany; 3 Department of Neurology, Faculty of Medicine, RWTH Aachen University, Aachen, Germany; 4 Department of Psychiatry, Psychotherapy and Psychosomatics, RWTH Aachen University, Aachen, Germany; 5 Institute of Neuroscience and Medicine 11, INM-11, JARA, Forschungszentrum Jülich, Jülich, Germany; University of Mississippi, UNITED STATES

## Abstract

**Objective:**

Quantitative values derived from PET brain images are of high interest for neuroscientific applications. Insufficient DT correction (DTC) can lead to a systematic bias of the output parameters obtained by a detailed analysis of the time activity curves (TACs). The DTC method currently used for the Siemens 3T MR BrainPET insert is global, i.e., differences in DT losses between detector blocks are not considered, leading to inaccurate DTC and, consequently, to inaccurate measurements masked by a bias. However, following careful evaluation with phantom measurements, a new block-pairwise DTC method has demonstrated a higher degree of accuracy compared to the global DTC method.

**Approach:**

Differences between the global and the block-pairwise DTC method were studied in this work by applying several radioactive tracers. We evaluated the impact on [^11^C]ABP688, O-(2-[^18^F]fluoroethyl)-L-tyrosine (FET), and [^15^O]H_2_O TACs.

**Results:**

For [^11^C]ABP688, a relevant bias of between -0.0034 and -0.0053 ml/ (cm^3^ • min) was found in all studied brain regions for the volume of distribution (V_T_) when using the current global DTC method. For [^18^F]FET-PET, differences of up to 10% were observed in the tumor-to-brain ratio (TBR_max_), these differences depend on the radial distance of the maximum from the PET isocenter. For [^15^O]H_2_O, differences between +4% and -7% were observed in the GM region. Average biases of -4.58%, -3.2%, and -1.2% for the regional cerebral blood flow (CBF (K_1_)), the rate constant k_2_, and the volume of distribution V_T_ were observed, respectively. Conversely, in the white matter region, average biases of -4.9%, -7.0%, and 3.8% were observed for CBF (K_1_), k_2_, and V_T_, respectively.

**Conclusion:**

The bias introduced by the global DTC method leads to an overestimation in the studied quantitative parameters for all applications compared to the block-pairwise method.

**Significance:**

The observed differences between the two DTC methods are particularly relevant for research applications in neuroscientific studies as they affect the accuracy of quantitative Brain PET images.

## Introduction

**Fig 1:**
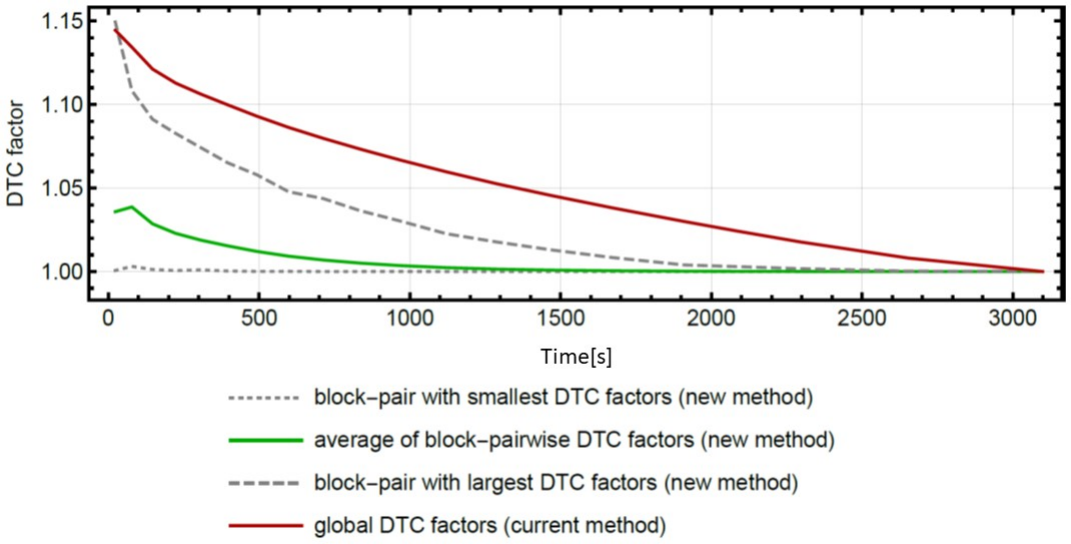
The differences in the DTC factors for both DTC methods for a typical data set. Data were obtained from a typical [^11^C]ABP688 volunteer measurement.

PET imaging is an important tool for the diagnosis of brain diseases, and since PET is able to measure the concentration of injected radiotracers in a quantitative way, it is an ideal tool for the quantification of (patho-) physiological functions of cerebral energy, neuroreceptor, and amino acid metabolism in vivo [1]. However, for this to be achieved, highly accurate data correction methods are needed. Consequently, the improvement of data acquisition and correction methods is particularly significant for assuring the quantification accuracy of PET imaging data. Data corrections, such as attenuation correction, decay correction, scatter correction, and dead time correction, are fundamental aspects of PET image reconstruction, and the continuous research undertaken to improve these corrections aims to increase the quantitative accuracy of PET imaging. The present study focuses on the improvement of dead time correction (DTC), as dead time (DT) losses affect the quantification of the reconstructed images considerably by causing an underestimation of the pixel values [2–4]. The main effect of the DT losses is the distortion of the recorded counts, which also affects the Poisson behavior [5–8]. The DT losses lead to an erroneous mean value of recorded counts and consequently to a systematic bias in the quantification of the (patho-) physiological functions to be investigated. Furthermore, DT losses can vary considerably during the scan time as the radiotracer distributes in the body and decays. This is particularly significant in dynamic studies, where varying DT losses would lead to time-dependent bias, especially in the case of short half-life radiotracers where the radioactivity changes most considerably between injection time and acquisition end [9, 10]. A more accurate DTC method decreases quantification biases in image reconstructions and results in more accurate mean counts [11–13]. Based on the method developed by [14], we recently developed an improved block-pairwise DTC method for the BrainPET insert of a 3T MRI scanner [15]. In this previous study, phantom measurements were used to quantitatively evaluate the accuracy and precision of the block-pairwise DTC method with respect to the differences between the reconstructed activity concentrations and true activity concentrations in the phantoms and phantom compartments. The results of the study showed that the block-pairwise DTC method was more accurate than the global DTC method currently used for examinations with the BrainPET. Furthermore, it was demonstrated that global DTC actually introduces a count-rate-dependent bias. However, a thorough comparative evaluation of the method in terms of the impact of this bias on the reconstructed PET images, its effect on typical quantitative magnitudes, and its dependence on different application schemes and radiopharmaceuticals was beyond the scope of the study. Therefore, this current work serves as an extension of the previous study by providing a careful analysis of the block-pairwise DTC method in terms of typical clinical and research applications. These applications differ significantly in their count rates measured over the entire scan time. For example, the count rates may vary due to the physical half-life of the radioisotopes used, the biodistribution of the radiopharmaceutical, and the application scheme, e.g., one bolus, several boli, or a bolus-infusion scheme. All of these influences can lead to different degrees of bias, whereby the severity of the bias may be highly relevant for the described applications. Furthermore, as detected count rates are also affected by inter-subject variations, the aforementioned DTC-related bias was assessed for different typical radiopharmaceuticals and applications by comparing identical reconstructions of the same datasets using the two different DTC methods. The global DTC method is dependent on the overall count rate, which is obtained by averaging over all individual scintillation detector blocks in the PET system, irrespective of the physical position of each block. Consequently, depending on the distribution of the activity inside and outside the FOV, quantification accuracy may be impaired since the global DTC applies the same correction factor to all detector blocks, although they receive different count rates [15]. In contrast, as the block-pairwise DTC method can obtain activity-independent and location-independent quantitation in phantom measurements, the mean of DT losses is estimated correctly [14, 15]. Furthermore, as previously shown [15] also analyzed the propagation of both the DTC methods into image noise, and the results showed that it was only slightly higher in the case of the block-pairwise DTC method. The dedicated BrainPET scanner used in this work is the Siemens 3T MR-BrainPET insert [16–19]. The insert fits inside the MR tunnel. The PET ring has no shields and has a significantly smaller diameter than that used in whole-body systems. Furthermore, it is subjected to activity not only from the inside field of view (FOV) but also from one end of the FOV (body activity outside of the FOV (oFOV)). The oFOV activity increases the number of events for detectors located closer to the patient’s side, which causes an uneven increase in DT and random coincidences [4]. Therefore, the homogeneity of the single rates in the detector blocks is affected, causing variations in the DT depending on the physical position of each detector block. According to earlier results [15], the block-pairwise DTC method is particularly beneficial in this respect and has shown consistent results for brain-sized phantoms inserted in the BrainPET, leading to improved quantification accuracy. This is due to the fact that it is based on the estimation of the DTC factor as a nonlinear function derived from the random coincidence rate in individual detector rings [14, 15]. Fig 1 shows the differences in the DTC factor for both DTC methods when applying them to data recorded in a typical measurement with the neuroreceptor ligand [^11^C]ABP688 on schizophrenic patients [20]. It can be observed that the global DTC factors (current method) lead to significantly higher correction factors, even when compared to the block-pair method with the largest DTC factors (new method). Furthermore, the global DTC factors (current method) actually lead to an overestimation of the activity concentration in the image [15]. This overcorrection of the DTC method also requires the comparison of the quantitative and semi-quantitative parameters obtained from the PET images. Further, as the calibration is normally done with phantom activities, which leads to significantly different DTC factors for both methods, both DTC methods need to be cross-calibrated with each other.

The main motivation of this work is to objectively compare the two DTC methods in terms of quantitation bias, as this factor can significantly impact clinical and research applications, leading to inaccurate results. The present work extends the previous study [15] by comparing the global and block-pairwise DTC methods for three different radiotracers with individual imaging protocols and quantitation methods. The investigated radiotracers are: [^11^C]ABP688, [^18^F]FET, and [^15^O]H_2_O. The studies with the glutaminergic neuroreceptor ligand [^11^C]ABP688 applied a bolus plus infusion protocol (BI) [20]. For this application, time activity curves (TACs) were evaluated, as well as effects on the binding potential (BP_ND_) and the volume of distribution (V_T_). Another set of data was obtained from a selection of brain tumor studies, for which the amino acid radiotracer [^18^F]FET [21, 22] was administered as a single bolus. In this case, the effects of the different DTC on TACs and mean and maximum tumor-to-brain ratios (TBR_max_ and TBR_mean_) were compared. Finally, the effect of the different DTC methods on TACs obtained from studies with repeated bolus injections of the cerebral blood flow (CBF (K_1_)) radiotracer [^15^O]H_2_O was evaluated. In this application, the regional CBF (K_1_), V_T_, and the rate constants k_2_ were compared with respect to the two DTC methods. The objective of this present study is to evaluate the impact of DTC bias on the aforementioned quantities. Other potential inaccuracies caused by factors other than the DTC were not considered.

## Methods

### Dead time correction method

**Fig 2:**
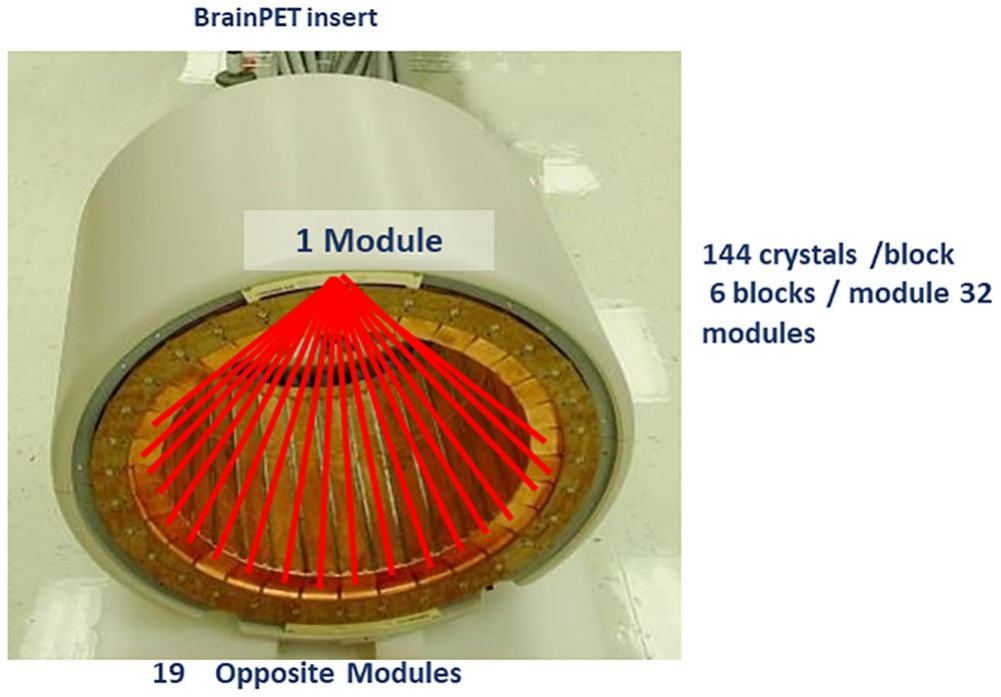
The Siemens 3T MR BrainPET insert detector together with the indication of the 19 accepted coincidences for one of the uppermost detector heads. Within an accepted head pair, coincidences between all 6 blocks of one head with all 6 blocks of the opposed head are allowed.

The DTC factors (*CF*_*DT*_) of the block-pairwise DTC were computed for all 10,944 block pairs (32 detector modules, each in coincidence with 19 opposed modules and each module having six independent blocks) using Eq (1) [15]. Fig 2 shows the Siemens 3T MR BrainPET insert together with the indication of the 19 accepted coincidences for one of the uppermost detector cassettes (modules). The total of (*CF*_*DT*_)s was then used to correct the prompt counts for all 10,944 block pairs. To further improve the accuracy of the dead time correction, the effect of random triple coincidences was also considered in this calculation. The parameters for the correction method were estimated by fitting the observed count rates with theoretical models of the expected count rates with and without DT losses. The block-wise single DT losses are well described by the non-paralyzable DT model (NPM) with an adaptation to account for the non-negligible natural background from the radioactive lutetium isotope in the scintillation crystals.

The block-pairwise DTC method is based on the observation that DT losses of the prompt coincidence events can be estimated from the DT losses of the delayed coincidence events of the corresponding block pair. A decay experiment with a phantom was used to calibrate and validate the method, as previously shown [15].

The DTC factors for the individual block pairs were computed using this model:

$$CF_{DT}=\;\frac{{\left(O_{R_{ideal}}-\;\frac{A_{R_{ideal}}\;O_{R_{ob}}}{A_{R_{ob}}}+\;\frac{A_{R_{ideal}}\;{\left(\frac{R_{ob}}{m}\right)}^{k}}{\;\sqrt{A_{R_{ob}}^{2}{\left(\frac{R_{ob}}{m}\right)}^{k}\left(1-\;\;\tau {\left(\frac{R_{ob}}{m}\right)}^{\;k}\right)}}\right)}^{2}}{R_{ob}},$$ (1)

where $O_{R_{ideal}}$ is introduced as the independent pedestal for DT loss-free delayed random coincidences. $A_{R_{ideal}}$ is given by $A_{R_{ideal}}=\varepsilon ∙{A'}_{R_{ideal}}$, where ${A'}_{R_{ideal}}$ is the tracer activity in the phantom at *t* = 0 for DT loss-free delayed random coincidences and *ε* is the detection efficiency for the delayed random coincidences. $O_{R_{ob}}$ is the corresponding pedestal accounting for natural background radiation. $A_{R_{ob}}$ is given by $A_{R_{ob}}=\varepsilon ∙{A'}_{R_{ob}}$, where ${A'}_{R_{ob}}$ is the tracer activity in the phantom at *t* = 0. *R*_*ob*_ is the observed delayed random coincidences rate due to triples, *m* and *k* are empirical fit parameters, and *τ* is the DT constant of the corresponding block pair. The values for these parameters have been previously determined during the calibration of the method, as previously shown [15].

### Data acquisition

All in vivo measurements were performed with a Siemens 3T MR BrainPET insert, which is a dedicated head PET insert for use with the Siemens 3T MAGNETOM TIM Trio MRI scanner with an adapted head transmit-receive (Tx/Rx) coil. The Tx/Rx coil fits into the BrainPET and consists of a single channel transmit-receive coil and an 8-channel receive coil [19]. The BrainPET insert uses avalanche photodiodes (APDs) and cerium-doped lutetium oxyorthosilicate (LSO) as the scintillator [23]. Scintillation event processing was done on the Quicksilver platform [24, 25]. The data were recorded in list mode [26, 27] and then binned into sinogram format. The energy acceptance window was set to 420–600 keV. The random coincidences were estimated by the delayed window technique [3, 27]. The coincidence time window was 12 ns. The PET scanner consists of 192 detector blocks arranged in 32 modules (heads), each module bearing six detector blocks. Each detector block has 12 x12 scintillation pixels connected by a light guide to a 3 x 3 array of Hamamatsu S8664–55 APDs, each with a sensitive area of 5 x 5 mm^2^. The scintillation detector blocks are attached to readout electronics for position and energy estimation, coincidence detection, and random coincidence estimation. The LSO crystals have a pixel pitch of 2.5 mm x 2.5 mm and are 20 mm long. The BrainPET insert has an axial FOV of 19.2 cm and a transversal FOV of 31.4 cm [19, 28–30].

### Cross-calibration

To relate the reconstructed image voxel values to the actual activity concentration in the corresponding tissue region, a cross-calibration factor (CCF) was applied. As we have previously shown that the bias of the global DTC method propagates into the calibration factor, the two DT correction methods considered in this work necessarily lead to different calibration factors [15]. In order to convert the reconstructed count rate images into an image of activity concentration, the calibration factor has to be applied after the image reconstruction with all corrections. Instead of determining the calibration factors for both methods, a CCF between the global and the block-pairwise DTC method can be determined alternatively. For this purpose, we used a homogenous cylinder with an inner diameter of 14 cm and a length of 23.6 cm (volume: ≈ 3633 ml) filled with 53.7 MBq ^18^F diluted in water. The phantom was transversely centered in the FOV. The phantom was axillary centered to the extent that the dimensions of the MR transmit/receive head coil allowed. The acquisition lasted for 20 minutes. A CCF of 1.08 was obtained as the ratio between the calibration factors for the block-pairwise and the global DTC method. The CCF is time-independent and a scalar factor for the entire PET system. It was applied to all reconstructed images using the block-pairwise method.

### Measurements

PET data from three different radiotracers administered in previous measurements with healthy volunteers and patients were used to compare the DTC methods. The first set of measurements pertained to [^11^C]ABP688, a glutaminergic neuroreceptor ligand, which has been previously used to investigate mGluR5 binding with and without a cognitive task [20]. For the present study, data sets from eight subjects in the [^11^C]ABP688 cohort were chosen. All subjects were male and between 24 and 50 years of age. Four of them were smokers, and four were non-smokers. Six subjects were healthy volunteers, while two were diagnosed with schizophrenia.

The [^11^C]ABP688 data were collected between 2017 and 2019; the patients were recruited by Uniklinik RWTH Aachen, and the study was approved by the Ethics Committee of the Medical Faculty at the RWTH Aachen University and the German Federal Office for Radiation Protection (Bundesamt für Strahlenschutz). The procedures adhered to the standards established in the Declaration of Helsinki. All subjects gave prior written, informed consent for their participation [20]. The [^11^C]ABP688 acquisition took 65 minutes, starting with a bolus injection followed by a constant infusion. This bolus-infusion (BI) protocol was optimized by injecting around 50% of the total activity with the bolus, followed by the infusion of the remaining activity at a rate of 92 ml/h. Decay-corrected PET data reached equilibrium 30 minutes after the bolus injection. After reaching equilibrium, participants had to complete an auditory cognitive task during the MR-PET scan [20]. The average total administered activity per subject was 525 ± 55 MBq [20]. The [^11^C]ABP688 data were used to compare the BI and TACs, and the derived quantitative parameters, such as the BP_ND_ and the V_T_. Table 1 summarizes the details of the [^11^C]ABP688 cohort used in this study.

**Table 1:** Details of the [^11^C]ABP688 study volunteer cohort.

Subject #	Age (y)	Sex	smoker/non-smoker	Healthy volunteer/Patient
				(Initial Diagnosis)
**1**	24	m	non-smoker	Healthy volunteer
**2**	49	m	smoker	Healthy volunteer
**3**	50	m	smoker	Healthy volunteer
**4**	37	m	non-smoker	Healthy volunteer
**5**	25	m	non-smoker	Healthy volunteer
**6**	47	m	smoker	Healthy volunteer
**7**	26	m	non-smoker	Patient (schizophrenia)
**8**	45	m	smoker	Patient (schizophrenia)

For the [^11^C]ABP688 measurements, 13 venous blood (arterialized) samples of 5 ml were taken at 2,5,10,15,20,25,30,35,40,45,50,55 and 60 minutes after the bolus injection. The processing of the blood samples is described in [31].

The second data set consisted of twenty O-(2-[^18^F] fluoroethyl)-L-tyrosine ([^18^F]FET) measurements from a cohort of patients with histologically confirmed brain tumors. These data were used to compare the influence of the developed DTC on the TAC curve shape and semi-quantitative parameters, such as TBR_mean_ and TBR_max_ [32]. The dynamic PET data were acquired between 0 and 50 minutes after injection (p.i.) of a [^18^F]FET bolus. The average injected radioactivity was 220 ± 32 MBq. All measurements were conducted between 2018 and 2021. The static and dynamic [^18^F]FET PET parameters were calculated. The age range of this cohort was (26–69 years), with nine females and 11 male subjects. All [^18^F]FET measurements were approved by the local ethics committee of the RWTH Aachen University Hospital, and the procedures adhered to the standards established in the Declaration of Helsinki. All subjects gave prior written, informed consent for their participation [33]. Detailed [^18^F]FET patient cohort characteristics are summarized in Table 2.

**Table 2:** Detailed [^18^F]FET patient cohort characteristics.

Measurements #	Age (y)	Sex	Initial Diagnosis	WHO grade	IDH Genotype
**1**	59	f	Glioblastoma	IV	wildtype
**2**	67	m	Oligodendroglioma	III	mutant
**3**	59	m	Glioblastoma	IV	wildtype
**4**	35	f	Glioblastoma	IV	wildtype
**5**	49	m	Oligodendroglioma	II	mutant
**6**	26	m	Astrocytoma	III	mutant
**7**	39	m	Glioblastoma	IV	wildtype
**8**	48	m	Glioblastoma	IV	wildtype
**9**	56	m	Glioblastoma	IV	wildtype
**10**	62	f	Glioblastoma	IV	wildtype
**11**	50	f	Oligodendroglioma	II	mutant
**12**	44	m	Oligodendroglioma	III	mutant
**13**	55	f	Brain Metastases	-	-
**14**	54	f	Oligoastrocytoma	II	n.a.
**15**	54	m	Oligoastrocytoma	II	n.a.
**16**	69	m	Glioblastoma	IV	wildtype
**17**	44	f	Glioblastoma	IV	wildtype
**18**	50	m	Oligodendroglioma	II	mutant
**19**	60	f	Suspected Glioma	unknown	unknown
**20**	44	f	Glioblastoma	IV	wildtype

n.a. = not available

The third data set consisted of four [^15^O]H_2_O data sets collected in 2012 for a study on cerebral blood flow [34]. The protocol for this study was designed to incorporate a task that had to be completed by the volunteers under different states of attention. In this original work, each subject received four [^15^O]H_2_O injections of 536.75 ± 2 MBq [34]. Each volunteer was measured on two different days (four bolus applications during each of these measurements), once after a night with sleep deprivation and once after a night without sleep deprivation. For each of the [^15^O]H_2_O injections, 180 seconds of PET data were acquired, and the time between subsequent injections on the same day was always > 15 minutes (seven half-lives of ^15^O) to ensure the residual activity from the previous bolus to be smaller than 1%. The data analyzed was taken from the examinations without sleep deprivation. The third and fourth acquisitions with [^15^O]H_2_O were obtained from the same volunteers in successive measurements. The acquisition time was 180 seconds, data were acquired in list mode, four injections were administered during each measurement, and 180 seconds were used for reconstruction following each injection [34]. All the subjects were males between 27 and 31 years. The [^15^O]H_2_O data were used to compare CBF (K_1_) and V_T_. The rate constant k_2_ was compared in relation to the two DTC methods.

In this study, the model flow and dispersion from PMOD were utilized to estimate dispersion and delay, and corresponding corrections for it were included in the one-tissue compartment model for the estimation of CBF (K_1_). Consequently, based on the information obtained from the initial modeling/fitting, the dispersion-corrected curve for blood and the delay were applied in the traditional one-tissue compartment model from PMOD, where CBF (K_1_), k_2_, and V_T_ were automatically generated.

Table 3 resumes [^15^O]H_2_O measurement details for all volunteers. The study was approved by the ethics committee of the university hospital of the RWTH University Aachen and the federal authorities according to the Declaration of Helsinki’s Ethical Principles for Medical Research Involving Human Subjects and the German radiation protection law. All participants gave prior written approval.

**Table 3:** The [^15^O] water PET measurements data.

Measurements #	Injection #	Age (y)	Sex	Injection time (relative to acquisition start time)	Acquisition start time on the BrianPET scanner	Activity [MBq]	Subject (new/follow through)	Sleep deprivation (with/without)
**Measurement** 1	1	27	m	00:04:05	12:49:51	537	New subject	with
	2			00:03:27	13:10:09	536		
	3			00:03:15	13:41:56	537		
	4			00:02:37	14:00:38	537		
**Measurement** 2	1	31	m	00:43:33	11:37:04	538	New subject	Without
	2			00:04:10	12:06:28	536		
	3			00:04:43	12:27:04	537		
	4			00:04:26	12:45:59	537		
**Measurement** 3	1	29	m	00:03:11	10:14:32	538	New subject	With
	2			00:03:19	10:31:42	538		
	3			00:03:45	10:50:24	538		
	4			00:03:59	11:08:48	537		
**Measurement** 4	1	29	m	00:03:28	09:33:30	535	Follow through of same subject as in Measurement **#** 3	Without
	2			00:04:51	09:53:04	536		
	3			00:04:45	10:13:12	535		
	4			00:04:33	10:33:28	536		

The image acquisitions and raw data used in this study were previously obtained for use in other studies but have not been publicly disclosed. Specifically, only the phantom measurements were obtained for this study, and no additional volunteer or patient data were collected. Ethical approval encompassed both patient and volunteer data acquired in prior studies, permitting their reuse for further research at Forschungszentrum Jülich GmbH (FZJ). No data access committee participated in this study. While the raw data and image acquisitions can be accessed within Forschungszentrum Jülich GmbH (FZJ), they cannot be made publicly available due to data privacy legislation.

### Data analysis

The DT correction was carried out with both the current method (global DTC) and the new method (block-pairwise DTC) and applied during the image reconstruction for all mentioned measurements. That is, all data sets were subjected to two separate reconstructions, with the only variation being the DTC correction employed. All other processing steps, such as image reconstruction, framing, random correction, decay correction, attenuation correction, scatter correction, and any potential other steps, were applied uniformly to both reconstructions. This approach ensured that a pair of directly comparable reconstructed images could be obtained for each PET data set, even in the absence of the ground truth, which is common for volunteer images. This methodology enables the exact quantification of the impact that the two DTC methods have on the reconstructed activity concentrations, as well as on any of the aforementioned derived quantities, since the PET reconstruction is deterministic for repeated reconstructions of any identical data set. In addition, any observed difference between both reconstructions of the same data set can only be attributed to the different DTC methods. All images were reconstructed using the 3D OP-OSEM algorithm with two subsets and 32 iterations [35, 36]. The image volume was 153 x 256 x 256 voxels with an isotropic voxel size of 1.25 mm^3^. In addition to the DTC, the data sets were corrected for decay, randoms, attenuation [37], and scatter. As mentioned above, the CCF was obtained from the measurement with a cylindrical phantom filled with ^18^F and located inside of PET FOV. Single cylindrical ROIs were used for the analysis. A cylindrical ROI was placed in the center of the phantom images and corrected with the global and block-pairwise DTC. The ROI dimensions of x ≈ 98 mm, y ≈ 97 mm, and z ≈ 125 mm covered most of the phantom area (in the x and y-axis). The (tangential) border of the ROI was 1 cm from the edges. The ROIs were aligned at the scanner axis for all image frames of 20 minutes in length. The mean activity concentration was estimated for the block-pairwise DTC method and further compared to the mean activity concentration of the global DTC method to obtain the CCF for the images reconstructed with the block-pairwise DTC. The analysis was performed using AMIDE (Amide’s Medical Imaging Data Examiner software) [38].

For the [^11^C]ABP688 measurement, we applied the constant true coincidence count rate framing scheme developed previously to minimize reconstruction bias at low counts [13, 15, 39]. The [^11^C]ABP688 images were analyzed with PMOD (version 4.103, PMOD Technologies, Zurich, Switzerland, now Bruker) and the PNEURO package. The images were post-processed using a 3D Gaussian post-reconstruction filter (2.5 mm). Furthermore, they were corrected for motion, normalized by the stereotactic normalization of PMOD (version 4.103, PMOD Technologies, Zurich, Switzerland, now Bruker), and matched to the simultaneously acquired MR T1 MPARGE image. All reconstructed images were normalized to the Montreal Neurological Institute (MNI) space and the Hammers Atlas [40]. The ROIs were drawn using the T1 MPARGE images as an anatomical reference. Three exemplary regions of the human brain were chosen for the analysis based on their relevance: cerebellum gray matter (GM), temporal posterior cortices, and the anterior cingulate cortex (ACC). The cerebellum GM was used as a reference region for the [^11^C]ABP688 study [39, 41]. A detailed description of [^11^C]ABP688 measurements can be found in [13, 42]. We evaluated the [^11^C]ABP688 TACs and derived quantities, i.e., BP_ND_ and the V_T_ [43, 44]. The BP_ND_ was computed by dividing the mean activity concentration in the target region by the mean activity concentration in the reference region, i.e., the cerebellum GM, and subsequently subtracting 1.0. The V_T_ was computed by building the ratio of the tracer concentration in the target tissue to the concentration in the plasma (metabolite correction was applied) at equilibrium [12, 43, 44]. The bias change was parameterized using linear approximation. This was achieved by computing the slope of the V_T_ and the BP_ND_ during the cognitive task phase (after reaching equilibrium without the cognitive task, i.e. t > 30 min.) using linear regression. This interval is especially relevant for assessing if the equilibrium condition for the tracer is fulfilled. If the equilibrium is reached, the slope of the V_T_ vs. time in the corresponding interval must be 0. Any slope introduced by the DTC method would potentially mask the real slope of the V_T_ and the BP_ND_ during the neuroscientific experiment, leading to incorrect conclusions.

The dynamic data set of [^18^F]FET images was reconstructed using 16 frames of 5×1 minutes, 5×3 minutes, and 6×5 minutes. Summed images from 20–40 minutes p.i. were used, and standard uptake values (SUV) were obtained by dividing the radioactivity concentration (kBq/mL) in the tissue by the ratio of the injected radioactivity and the body weight (in kg). A 3D Gaussian filter (2.5 mm) was applied to all images, and the PET data set was corrected for motion and normalized. The uptake values were converted to SUV. The analysis of the [^18^F]FET images was done with PMOD (version 4.103, PMOD Technologies, Zurich, Switzerland). A spherical volume of interest (VOI) of constant size (diameter of 15 mm) was drawn at the contralateral side of the tumor area as a healthy reference. The 3D segmentation with PMOD was used to delineate the tumor volume by assigning all voxels with a TBR of 1.6 or higher to the tumor volume [45]. The shapes of the TACs derived from the reconstructed images were evaluated by curve fitting using a linearized TAC model designed for [^18^F]FET. This method was chosen as it has been shown that the shape of the time-activity curves correlates with the malignancy of the gliomas and that it also provides relevant information relating to the differentiation of treatment-related changes from tumor recurrence [32, 46, 47]. As TBR_max_ and TBR_mean_ are used for diagnosis, we computed these parameters for the period between 20 and 40 minutes p.i. for all cases. The TBR_max_ was calculated by dividing the maximum of the SUV of the tumor VOI (which was created by the threshold mask in PMOD) by the mean of the SUV of the healthy tissue VOI. The number of voxels for the peak area was fixed to 1021 voxels using the Max VOI tool in PMOD (mean uptake of 2 cm sphere centered at maximum voxel in tumor VOI) [48]. The TBR_mean_ was calculated by dividing the mean SUV of the tumor VOI by the mean SUV of the reference (healthy tissue) VOI. Mean relative differences in TBR_max_ and TBR_mean_ between the block-pairwise and global DTC were computed, and a correlation with the tumor size and the distance of the tumor with respect to the brain center point was tested. The distance was obtained with the data inspector tool in the PMOD from the mid-center point of the brain to the center of the peak (VOI) of the tumor area. The tumor size was obtained by the ISO-contouring VOI tool at a threshold level relative to uptake in PMOD (version 4.103, PMOD Technologies, Zurich, Switzerland). As not all values were distributed normally, the Spearman-Rank correlation test was used instead of the Pearson’s correlation test.

The list mode data of the [^15^O]H_2_O measurements were also framed using the constant true coincidence count rate framing schemes for stabilizing and minimizing reconstruction bias [13, 15, 39]. The data analysis was done with PMOD (version 4.103, PMOD Technologies, Zurich, Switzerland). A 3D Gaussian post-reconstruction filter (4 mm) was applied to the reconstructed images. The VOIs covering the GM and the WM were drawn manually for each subject with sufficient distance from tissue borders in order to minimize partial volume effects (PVE) [49, 50] and in order to allow validation against results obtained in the original work [34]. Furthermore, arterial blood data were corrected for dispersion and delay, as in the original work [34]. The measured TACs and the blood data were used to compute the CBF (K_1_), the rate constant k_2_, and the V_T_ via kinetic modeling [12, 51]. The kinetic modeling was done with PMOD (version 4.103, PMOD Technologies, Zurich, Switzerland), assuming a one-tissue compartment model. The CCF was applied to all reconstructed data sets when using the block-pairwise DTC method. Relative differences in the studied quantitative parameters were computed according to the following equation:

$$\text{rel}.\text{ diff}=100\text{\%}\;\;\frac{{\text{Q}}_{\text{block}-\text{pairwise}}-{\text{Q}}_{\text{global}}}{{\text{Q}}_{\text{global}}},$$ (2)

where Q_(block-pairwise)_ and Q_global_ refer to the studied quantitative PET parameters, e.g., BP_ND_, V_T_, CBF (K_1_), k_2_, TBR_max_, and TBR_mean_. These were obtained with the block-pairwise DTC method and the global DTC method.

## Results

**Fig 3:**
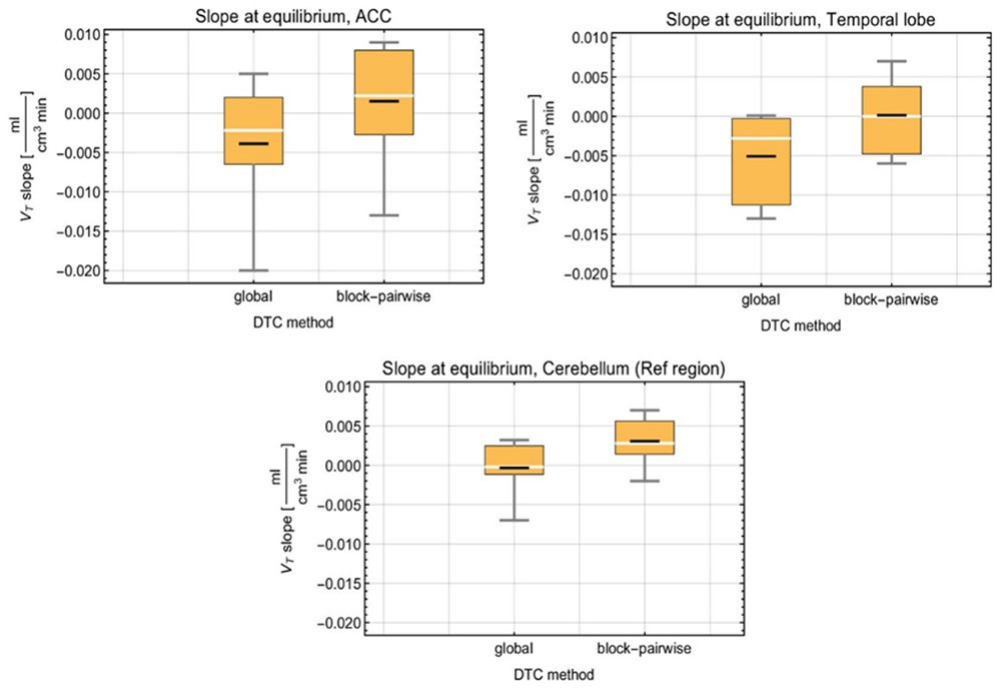
Statistical distribution of the slope values for V_T_ and both DTC methods for three exemplary brain regions obtained with the 3T MR-BrainPET insert. Black line: mean, white line: median, yellow box 25/75% quantile, fences: min/max values.

The evaluation of the [^11^C]ABP688 measurements showed that the global DTC method introduced a relevant bias in V_T_ when used with the Siemens 3T MR BrainPET insert for the studied brain regions. The bias was considerably smaller for BP_ND_, which was computed using the simple ratio method in this study because the DTC-caused bias is largely canceled out due to the quotient. Fig 3 compares the distributions of the V_T_ slopes in the three exemplary brain regions and within the scan time interval from approximately 30 minutes p.i. until the end of the scan. In the ACC region, the mean V_T_ slope was -0.0038 ml/ (cm^3^ x min) for the global DTC and 0.0015 ml/ (cm^3^ x min) for the block-pairwise DTC. In the temporal posterior cortices, the mean V_T_ slope was -0.0051 ml/(cm^3^ x min) for the global DTC, while it was 0.00014 ml/(cm^3^ x min) for the block-pairwise DTC. These results show relevant differences in the V_T_ values between both DTC methods during the time interval after reaching the equilibrium. Further, we observed different slopes in the ACC and the temporal posterior cortices. In the cerebellum GM (reference region), the mean V_T_ slope was -0.00033 ml/(cm^3^ x min) for the global DTC and 0.0031 ml/(cm^3^ x min) for the block-pairwise DTC. The mean V_T_ slopes obtained with global DTC are apparently closer to the ideal equilibrium, i.e. a slope of exactly 0 ml/ (cm^3^ x min), which can be explained by the fact that the BI scheme was optimized based on reconstructions using the global DTC method, which introduces a bias. This was shown in our previous work using phantom measurements [15].

**Fig 4:**
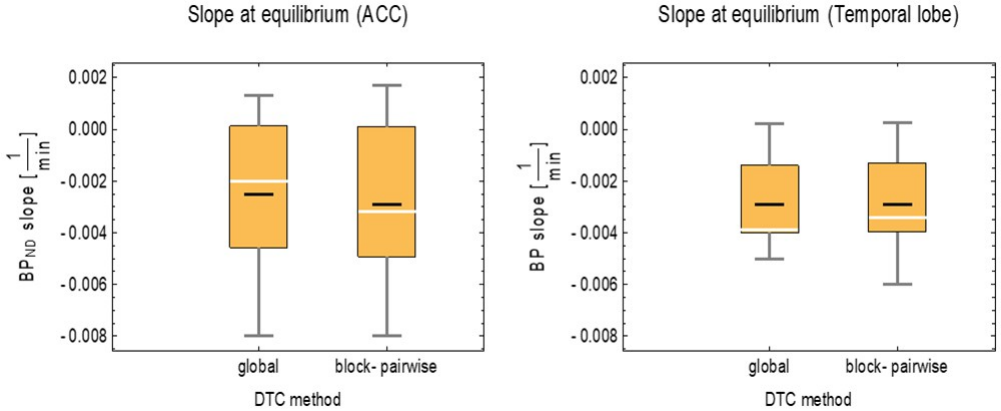
Statistical distribution of the slope values for BP_ND_ and both DTC methods for three exemplary brain regions obtained with the 3T MR-BrainPET insert. Black line: mean, white line: median, yellow box 25/75% quantile, fences: min/max values.

**Fig 5:**
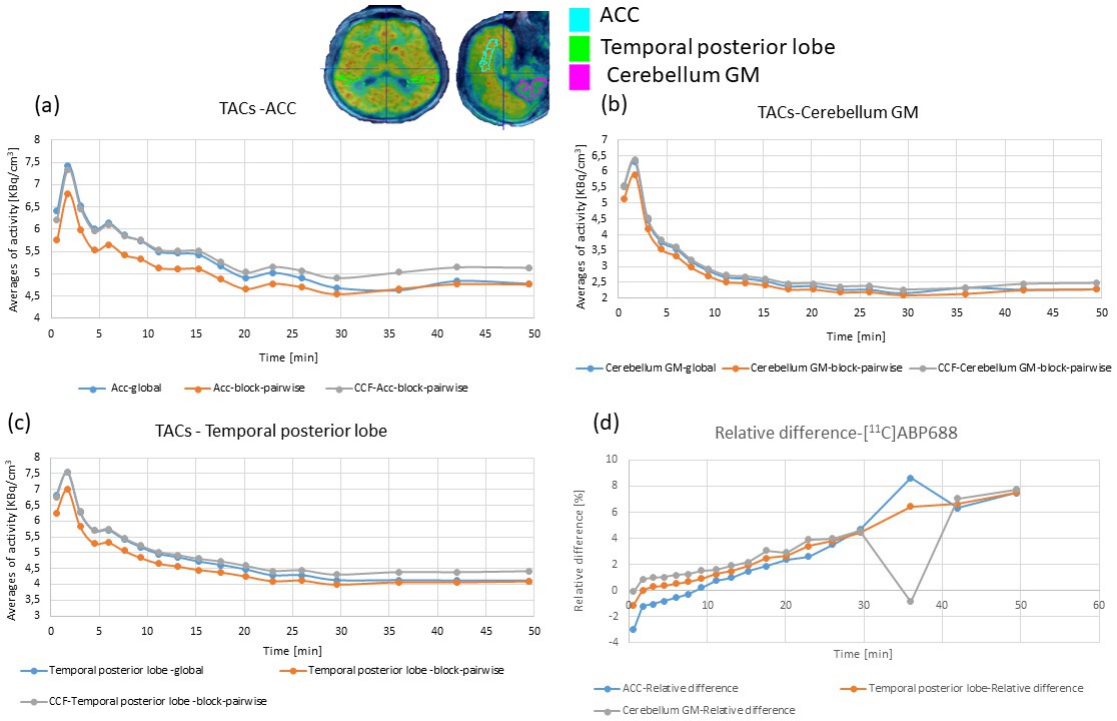
[^11^C]ABP688 TACs in three relevant brain regions for both DTC methods: (a) ACC, (b) the cerebellum GM, and (c) temporal posterior cortices. (d) shows the relative differences between both DTC methods. A CCF was applied to the reconstruction with the block-pairwise DTC method.

Fig 4 shows the distributions of the slope values of the BP_ND_ obtained for both DTC methods with the simple ratio method in two exemplary brain regions and for the scan interval from ≈ 30 min. p.i. to the end. In the ACC, the mean BP_ND_ slope was -0.0025 1/min for the global DTC and -0.003 1/min for the block-pairwise DTC. In the temporal posterior cortices, the mean BP_ND_ slope was -0.0029 1/min for the global DTC, while it was nearly the same with -0.00292 1/min for the block-pairwise DTC. The BP_ND_ did not show a real difference between either DTC method. Fig 5 shows the TACs in the three VOIs considered above for the [^11^C]ABP688 measurement processed with the two DTC methods and after applying the CCF to the reconstruction with the block-pairwise DTC method. The TACs obtained with the different DTC methods show clear differences in all three regions, and this difference can be seen to vary over the scanning time. However, as expected, the differences became smaller towards the end of the scan and after the steady-state was reached at around 25–35 minutes. Nevertheless, even a small difference in the TACs can lead to inaccuracy in the quantitative parameters obtained from the PET images. As previously shown, relative ratios between TAC values obtained with the block-pairwise DTC method and the global DTC method can differ from 1.4 to 1.1 in a non-linear manner in the course of the scan and are also strongly dependent on the ROI [15]. S1 Fig presents the relative errors for BP_ND_ and V_T_ averaged over all volunteer data sets, showing how the bias generated by the global DTC method changes at different time points during the scans (error bars represent the standard errors of the mean). Both S1 Fig and Fig 3 demonstrate that the equilibrium evaluation criterion and/or any task-related change in the BP_ND_ can be seriously masked by an inaccurate DTC.

**Fig 6:**
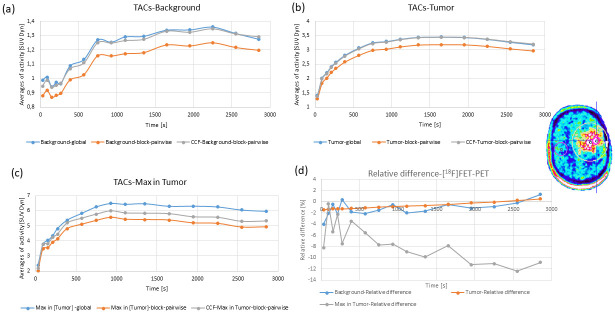
TACs obtained from one [^18^F]FET-PET image for three relevant types of VOIs for the global and the block-pairwise DTC methods. (a) Background VOI, (b) tumor VOI, and (c) max in tumor VOI. (d) shows the relative differences between both DTC methods. For the block-pairwise DTC method, TACs are shown before and after the application of the CCF.

The VOIs for the [^18^F]FET images were chosen to cover the background (healthy control tissue), the tumor volume, and the maximum concentration of the tracer in the tumor area. Fig 6 presents the TACs obtained from one [^18^F]FET image for three main VOIs with the global and block-pairwise DTC methods after the CCF has been applied to the block-pairwise DTC method. For the [^18^F]FET measurements, the example in Fig 6 shows that the global DTC method tends to overcorrect when compared to the block-pairwise DTC method in approximately 12% of the studied [^18^F]FET cases, even after applying the CCF. The overcorrection is considerable (average overcorrection was ≳ 7%) for the activity concentration inside the tumor-max VOI.

**Fig 7:**
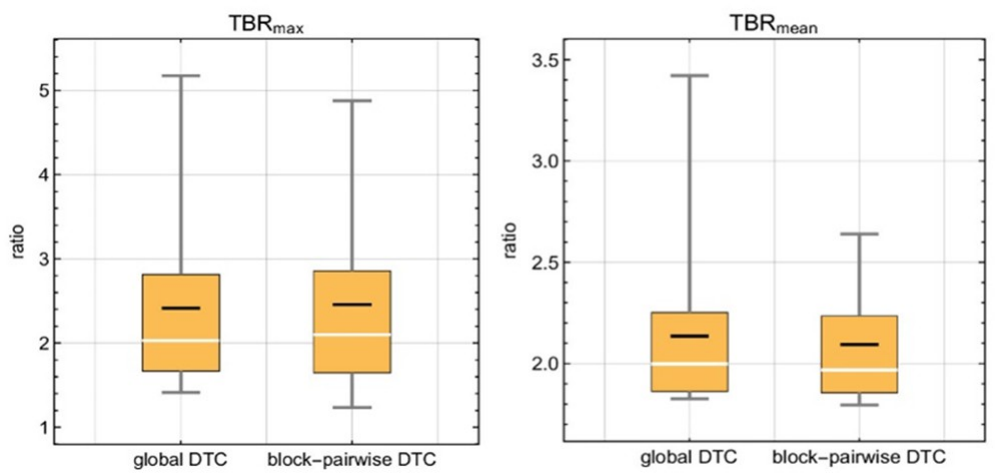
Statistics of differences between the global DTC method and the block-pairwise DTC method for relevant features of the [^18^F]FET-PET TBR_max_ and TBR_mean_.

**Fig 8:**
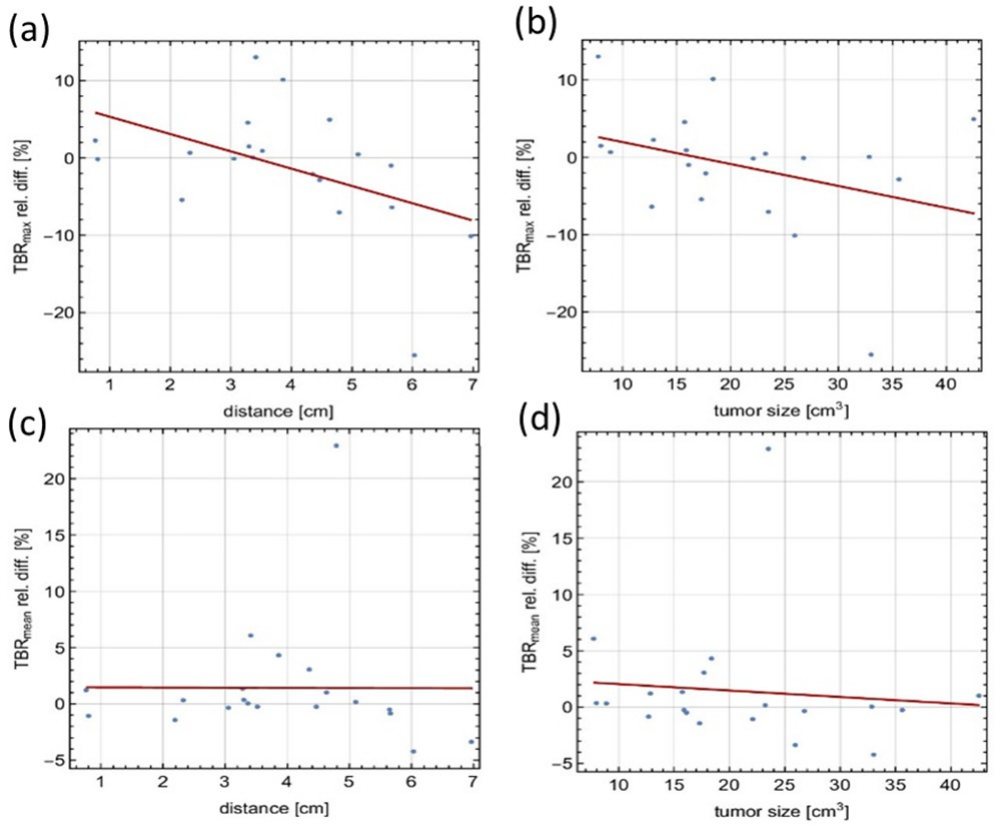
Dependency of the mean relative difference between both DTC methods in the time interval. (a) Dependency of the mean relative difference between both DTC methods in the time interval 20 to 40 minutes p.i. for TBR_max_ on the distance of the tumor to the PET FOV isocenter. (b) Dependency of the mean relative difference between both DTC methods in the time interval 20 to 40 minutes p.i. for TBR_max_ on the tumor size. (c) Dependency of the mean relative difference between both DTC methods in the time interval 20 to 40 minutes p.i. for TBR_mean_ on the distance of the tumor to the PET FOV isocenter. (d) Dependency of the mean relative difference between both DTC methods in the time interval 20 to 40 minutes p.i. for TBR_mean_ on the tumor size. Regression lines are shown in red.

Fig 7 shows the statistics for the differences in TBR_max_ and TBR_mean_ between the global DTC method and the block-pairwise DTC method for the time interval 20 to 40 minutes p.i. Fig 8 shows how the mean relative difference between both DTC methods in the time interval 20 to 40 minutes p.i. for the TBR_max_ and TBR_mean_ values depends on the tumor size and the distance of the tumor to the PET FOV isocenter. These plots indicate that the observed differences between global and block-pairwise DTC methods are strongly case-dependent for both TBR_max_ and TBR_mean_ and that the differences can be large. These differences are also heavily time-dependent in some cases. The relative differences of the averaged TBR_max_ and TBR_mean_ values over all twenty cases were around 4.4% between the global and the block-pairwise DTC methods.

The difference in TBR_max_ shows a significant dependency on the distance of the tumor from the isocenter at a significance level of 0.05 when all results are included. However, this result seems to be affected by a single data point (tumor distance from the isocenter of ≈ 6 cm, relative difference of ≈ -25%). In the correlation test without this data point, significance was no longer observed. The difference in TBR_max_ also depends on the tumor size, although not significantly. No dependency on tumor size or tumor distance to the isocenter was observed for the difference in TBR_mean_. In Table 4, Spearman rank test results for the dependencies in the TBR_max_ TBR_mean_ differences are resumed. S2 Fig shows the statistical distribution of the curve shape parameter obtained from various [^18^F]FET TACs and for the different DTC methods. The curve shape parameter casts the different behaviors of the [^18^F]FET TACs, i.e. rising, plateauing, and falling after reaching a peak, into a single shape parameter κ [32]. Previous studies have shown that the [^18^F]FET TAC shape is linked to the tumor grade [52–54].

**Table 4:** Spearman rank results for testing the correlation of differences in TBR_mean_ and TBR_max_ and tumor size and distance (data shown in Fig 8).

TBR_mean_ vs distance		TBR_mean_ vs size		TBR_max_ vs distance		TBR_max_ vs size	
Correlation factor	P-Value	Correlation factor	P-Value	Correlation factor	P-Value	Correlation factor	P-Value
-0.2	0.397873	-0.29173	0.212026	-0.46917	0.0368966	-0.38647	0.092342

**Fig 9:**
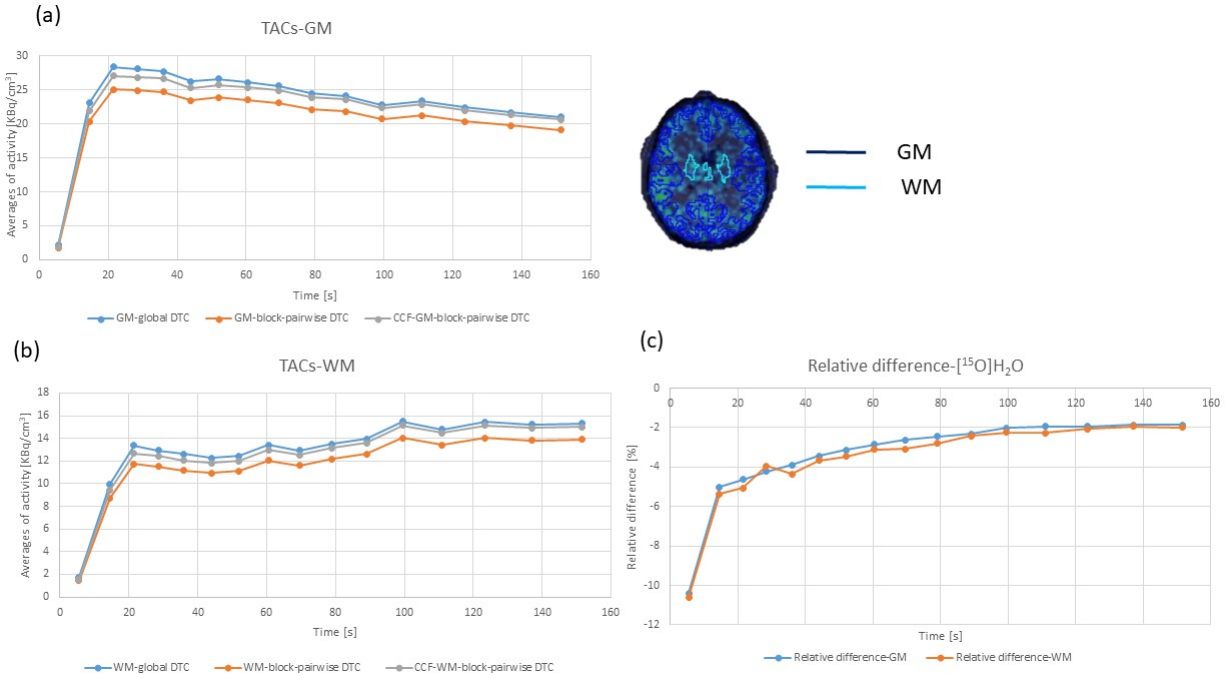
TACs and relative differences obtained from one [^15^O] water PET study for two relevant types of VOIs for the global and the block-pairwise DTC methods. For the block-pairwise DTC method, TACs are shown after the application of the CCF.

**Fig 10:**
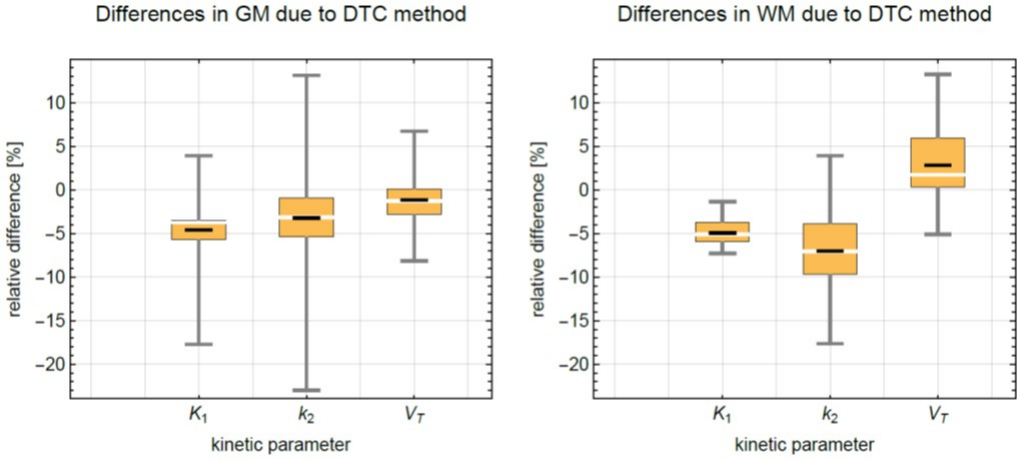
Statistical distribution of the relative differences for the kinetic parameters CBF (K_1_), k_2_, and V_T_ obtained from kinetic modeling of the [^15^O]H_2_O TACs assuming a one-tissue compartment model. Black line: mean, white line: median, yellow box 25/75% quantile, fences: min/max values. Left: GM, right: WM.

For [^15^O]H_2_O, Fig 9 shows the TACs obtained for two relevant types of VOIs, the GM and the WM, for the global and the block-pairwise DTC methods. For [^15^O]H_2_O, we observed mean relative differences for the studied kinetic parameters CBF (K_1_), k_2_, and V_T_ between +4% and -7%. Table 5 summarizes further statistical descriptors of the relative differences of the four kinetic parameters when comparing both DTC methods. We observed a small but noticeable bias in all four parameters CBF (K_1_), k_2_, and V_T_, for both regions. As the kinetic parameters obtained with the global DTC were considered as the reference when computing the relative differences, a negative difference corresponds to the case that the parameter obtained with the global DTC was smaller than the corresponding parameter obtained with block-pairwise DTC. Thus, with the exception of V_T_ in WM, values obtained with global DTC were always smaller by a few per cent. Plots of the statistical distributions of the relative differences are shown in Fig 10. The GM/WM ratios are close to 2.5 for both DTC methods.

**Table 5:** Main statistical descriptors of the relative differences of relevant kinetic parameters in GM and WM for of the [^15^O]H_2_O measurements.

Relative differences of bias values in GM [%]			Relative differences of bias values in WM [%]		
Parameter	Mean	StdDev	Parameter	Mean	StdDev
CBF (K_1_)	-4.58	4.6	CBF (K_1_)	-4.94	1.56
**k**_**2**_	-3.24	7.52	k_2_	-7	4.84
**V**_**T**_	-1.16	3.23	V_T_	3.83	4.73

GM: Gray Matter.

WM: White matter.

## Discussion

The presented evaluation is based on original work relating to improving DTC for the Siemens 3T MR BrainPET insert [15], where it was shown that the global DTC method currently used for the Siemens 3T MR BrainPET insert tends to overcorrect the DT losses compared to the block-pairwise method [15]. The present work aimed to examine the effects of block-pairwise DTC relative to global DTC on outcome parameters from three different human studies. In these studies, radiotracers with half-lives from 110 min to 2 min were administered with bolus injection and bolus plus infusion, respectively, thus leading to different dead time behavior. Those differences have been discussed to find their effects on the quantitative data. The glutaminergic neuroreceptor ligand [^11^C]ABP688 was examined exemplarily for the determination of the V_T_ data and BP_ND_ after reaching an equilibrium of the receptor ligand in the studied brain regions. The TACs for this application are characterized by rapidly changing activity in the PET FOV during the bolus phase and moderately changing activity in the PET FOV after reaching equilibrium. We observed relevant differences between the two DTC methods with respect to the slope of the distribution volume after reaching equilibrium. These differences were observed for all three studied regions and can be explained by the fact that for the determination of V_T_, the reconstructed image activity concentration is related to the activity concentration in the blood plasma, which itself is not affected by differences in either DTC method. However, when computing the BP_ND_ from the reconstructed image activity concentrations using the ratio between the target VOI and the cerebellum as the reference VOI, the differences occurring as a result of each DTC method cancel out to a large extent. Thus, the global DTC method introduces a small bias, which may mask both the equilibrium condition or deviations from this equilibrium in the case of cognitive tasks. The bias was especially observed for the determined V_T_, and moreover, the observed bias was different for the three regions. These differences must be expected for two reasons. First, the BI scheme does not guarantee that an equilibrium is reached in all brain regions [20, 55, 56].

Therefore, it seems that, on average, a slope of approximately 0 ml·cm^-3^·min^-1^ is reached in the cerebellum when using the global DTC method but not when using the block-pairwise DTC method. This is due to the fact that the BI scheme for the underlying study had been optimized before the availability of the block-pairwise DTC method [13]. Thus, the bias caused by the global DTC method also propagated into the data used for optimizing the BI scheme. In the temporal posterior cortices, a slope of approximately 0 ml·cm^-3^·min^-1^ is reached with the block-pairwise DTC method but not with the global DTC method. In the ACC, a slope of 0 ml·cm^-3^·min^-1^ is apparently not reached, neither for reconstruction with the global DTC nor for reconstructions with the block-pairwise DTC. The second reason for the observed behavior is the axial asymmetry of the dead time losses, which is caused by counts entering the FOV from the subject’s body. This leads to higher irradiation of the scintillation detectors at the frontal PET rings and consequently introduces a dependency of the DTC differences on the VOIs axial location. As can be seen in S1 Fig, even a small slope introduced by DTC in the time series of V_T_ and BP_ND_ values can impact the quantitative accuracy noticeably.

[^18^F]FET was used as an example of brain tumor imaging. [^18^F]FET TACs are characterized by a fast rising course after a bolus injection at the beginning of the PET scan, followed by a further rising or declining course towards the end of the scan. However, these TAC shapes lead to very comparable distributions of the shape parameter κ [32] for both DTC methods. Also, relevant diagnostic parameters such as the TBR_max_ and TBR_mean_ [21, 57] give rise to very comparable distributions when based on reconstructions with the two different DTC schemes, although rather large differences of up to 20% can be observed for individual cases. When considering the variations in the DTC methods with respect to tumor size and the distance of the tumor from the isocenter of the BrainPET insert, a discernable dependency becomes apparent. However, it is not significant. This observation can again be explained by the asymmetry in the dead time losses for individual detector blocks, which is larger the greater the distance of the tumor from the isocenter. In the case of TBR_mean_, these differences seem to effectively average out. As a consequence of these observations, it must be assumed that the global DTC method mainly increased the variance of TBR_max_ values.

CBF (K_1_) studies with [^15^O]H_2_O are accompanied by high count rates within a short acquisition period of 3 min, for example, and were used here to study the impact of the improved DTC on kinetic parameters obtained by modeling using a one-tissue compartment model. The TACs for this application are characterized by very fast-changing activity in the PET FOV over the entire scan time. A comparison between both DTC methods reveals that a small but consistent bias of 3–7% is introduced by the DTC for the four considered parameters, CBF (K_1_), k_2_, and V_T_, in both the GM region and the WM region. It should be noted that the sample size for all three data sets was relatively small. Consequently, the observed averaged bias values may change slightly when larger sample sizes are analyzed. Interestingly, this bias is an overestimation in both rate constants, K_1_ (CBF) and k_2_, in both GM and WM regions. This overestimation occurs despite the fact that the TACs are overestimated by the global DTC method, and one might expect an underestimation of the rate constants to compensate for the biased TACs from the one-tissue compartment model. Further, the size of the effect on CBF (K_1_), and k_2_ is slightly different in the studied regions, which translates into rather different V_T_ values. This is potentially due to the different influx/efflux behavior of both regions. This bias is an overestimation in all cases except for V_T_ in the WM region. Although the differences observed in the estimates CBF (K_1_), k_2_, and V_T_ were all <5% in GM and <7% in the WM, these differences can be exclusively attributed to the different DTC methods.

As the DTC introduced bias is count rate dependent, it also depends on the imaging protocol and the subject, leading to additional variation in the observed results for cohorts. Both effects can be mitigated by using the more accurate block-pairwise DTC method, thus also improving the quantitative accuracy of the previously mentioned kinetic parameters. This supports the conclusions made by Yamamoto et al. [14] and Freedman et al. [2].

Only very few studies have investigated DT effects, the majority of which have concentrated on SPECT rather than PET [58–61]. Thus, a comparison of the results is very difficult. Inoue et al. [58] showed that DT losses have a substantial effect on cerebral blood flow measurement when assessed by radionuclide angiography using SPECT and ^99m^Tc-HMPAO. The study showed that uncorrected DT count losses result in the overestimation of the calculated values in the TACs, thus leading to the conclusion that DTC has a high impact on quantification. Uribe et al. [59] investigated the impact of different DTC methods on ^177^Lu images obtained again with SPECT before and after radionuclide therapy. However, the DTC methods compared in this study did not differ in detail depending on if the global or block-wise DTC was used, but rather in the used energy acceptance window. Unfortunately, in this study, only the differences in the dead time correction factors are reported, and no patient-related quantities are given. Vicente et al. [60] evaluated an alternative DTC method for small animal PET using simulations and phantoms. Their main focus was to lower the accuracy dependency of the dead time correction on the activity level in the PET FOV, reporting a maximum deviation from the ground truth of 7%. We studied this previously for the evaluated block-pairwise method and found deviations from the ground truth to be smaller than 1.4% in all cases [15]. Cohalan et al. [61] also studied the impact of different DTC methods on ^177^Lu images obtained with SPECT in a phantom study. They compared a global DTC correction to a DTC for individual projections and reported relative quantitation differences in the range from 1% to 3%, which are comparable with our observations, although measured with a different modality. Freedman et al. [2] examined the distribution of singles rates of a PET scanner during cardiac studies with bolus injections of [^15^O]H_2_O and ^82^Rb and with slow infusion of [^18^F]FDG and static imaging studies. They came to the conclusion that the DT losses depend on the activity and that there were large differences in local single count rates. The study also showed that DTC methods that use spatially averaged DT loss determination (as in the case of the global DTC method examined in this study) can lead to inaccurate estimation of the absolute activity concentration, therefore creating regional errors.

## Conclusion

We evaluated the performance of an improved DTC method, i.e., the block-pairwise dead time correction method, by comparing it to the global DTC method with respect to the outcome parameters of three applications with different tracers, i.e., [^11^C]ABP688, [^18^F]FET, and [^15^O]H_2_O. The DTC factors obtained from both methods and their differences depend on the symmetry of the activity distribution related to the PET scanner geometry, i.e., the amount of activity from outside of the FOV and the activity distribution inside the PET FOV. For all applications, the global DTC method introduced a bias, e.g., an overcorrection, in the studied quantitative parameters, which was considerably reduced by the block-pairwise method. In the case of [^11^C]ABP688, The V_T_ slope was particularly affected by the bias, with differences of up to 0.005 ml·cm^-3^·min^-1^ between both DTC methods. For [^18^F]FET, differences in TBR_max_ of up to 10% were observed. In addition, these differences were dependent on the distance of the tumor from the PET isocenter. For [^15^O]H_2_O, we observed mean relative differences for the studied kinetic parameters CBF (K_1_), k_2_, and V_T_ of between +4% and -7%. With the exception of V_T_ in WM, all modeled parameters were overestimated when using the global DTC method. The observed differences and achieved improvements are highly relevant for research applications in neuroscientific studies as they affect the accuracy of the final quantification of the PET brain images.

## Supporting information

S1 Fig Relative differences averaged over all volunteer data sets obtained with [^11^C]ABP688 at different acquisition times (error bars represent the standard errors of the mean), indicating a time-dependent, DTC-introduced bias. (TIF)

S2 Fig Curve-shape parameter statistics for the FET-PET TACs and both DTC methods for three types of VOIs (i.e., healthy background volume, entire tumor volume, and maximum value volume). Black line: mean, white line: median, yellow box 25/75% quantile, whiskers: min/max values.
(TIF)

S3 Fig Scatter plots representing the slope values for V_T_ during equilibrium for the three exemplary brain regions and both compared DTC methods. The dashed line represents the identity.
(TIF)

S4 Fig Scatter plots representing the slope values for BP_ND_ during equilibrium for both exemplary brain regions and both compared DTC methods. The dashed line represents the identity.
(TIF)

S5 Fig Scatter plots representing the TBR_max_ and TBR_mean_ values for the [^18^F]FET-PET acquisitions for both compared DTC methods. The dashed line represents the identity.
(TIF)

S6 Fig Scatter plots representing the shape parameter of the individual [^18^F]FET-PET acquisitions for three types of VOI (i.e., healthy background volume, entire tumor volume, and maximum value volume). The dashed line represents the identity.
(TIF)

S7 Fig Scatter plots representing the rate constant K_1_ in GM and WM and both compared DTC methods. The dashed line represents the identity.
(TIF)

S8 Fig Scatter plots representing the rate constant k_2_ in GM and WM and both compared DTC methods. The dashed line represents the identity.
(TIF)

S9 Fig Scatter plots representing the distribution volume V_T_ in GM and WM and both compared DTC methods. The dashed line represents the identity.
(TIF)

S1 Data Values for the slope of V_T_ and BP_ND_ for both DTC methods for the three (two) exemplary brain regions, for the [^11^C]ABP688. The data is shown in Figs 3 & 4 in the manuscript and S3 & S4 Figs in the supporting data file.
(XLSX)

S2 Data This file contains the indiviudal measuremtns for TBR_max_ and TBR_mean_ and the curve-shape parameters for three types of VOIs (i.e., healthy background volume, entire tumor volume, and maximum value volume) and both DTC methods. These values are represented in Fig 7 in the manuscript and S2 Fig in the support file.
(XLSX)

S3 Data Values of the kinetic parameters CBF (K_1_), k_2_, and V_T_ and both DTC methods were obtained from kinetic modeling of the [^15^O]H_2_O acquisition assuming a one-tissue compartment model. These values are shown in Fig 9 and in Table 5 in the manuscript and plotted in figures: S7–S9 Figs in the supporting data file.
(XLSX)

S4 Data (XLSX)
