# Supplementary figures and images for: Impact of improved dead time correction on the quantification accuracy of a dedicated BrainPET scanner
Source: PLoS One. 2024 Apr 5;19(4):e0296357. doi: 10.1371/journal.pone.0296357 (PMC10997125; doi:10.1371/journal.pone.0296357)

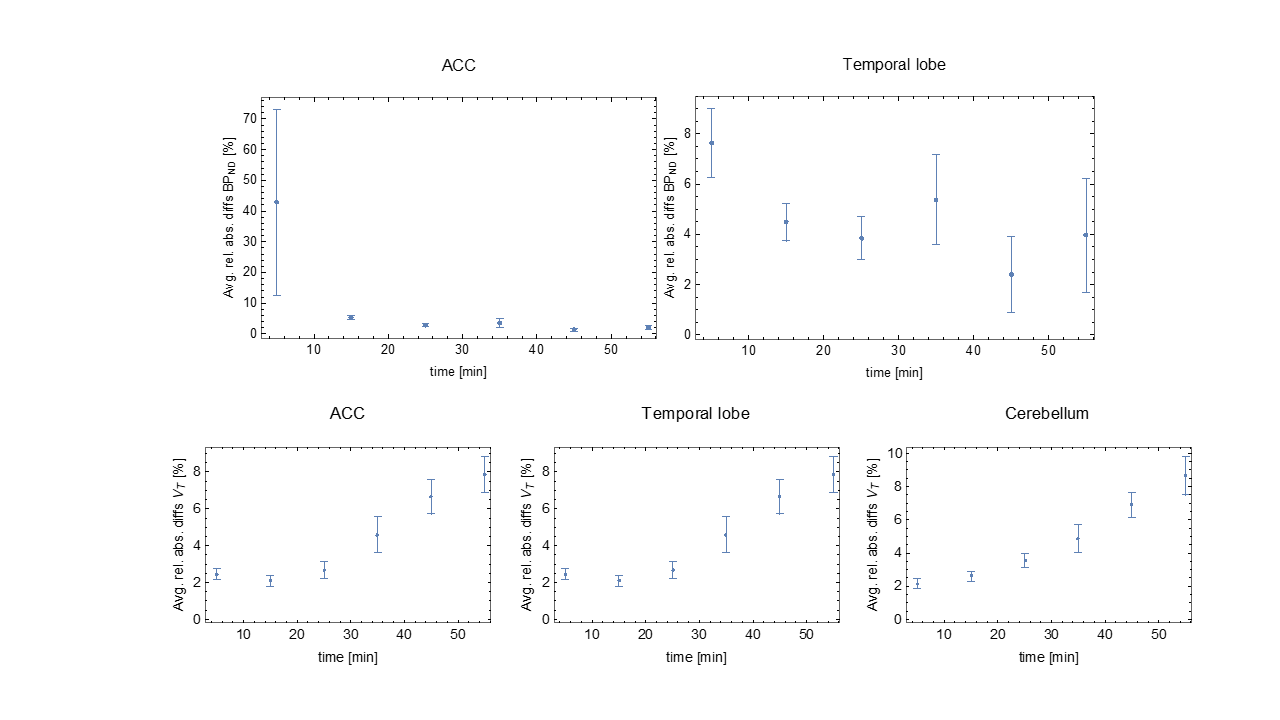

Supplement: S1 Fig — (TIF) [file pone.0296357.s001.tif]

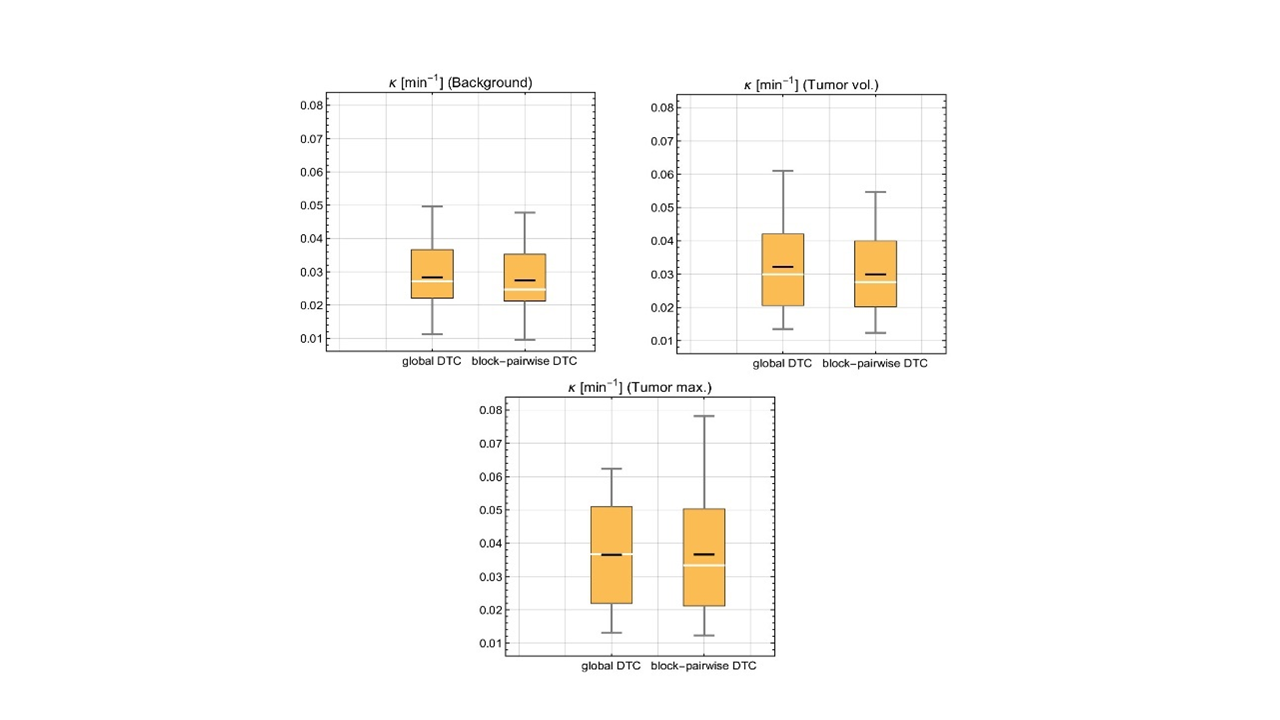

Supplement: S2 Fig — Black line: mean, white line: median, yellow box 25/75% quantile, whiskers: min/max values. (TIF) [file pone.0296357.s002.tif]

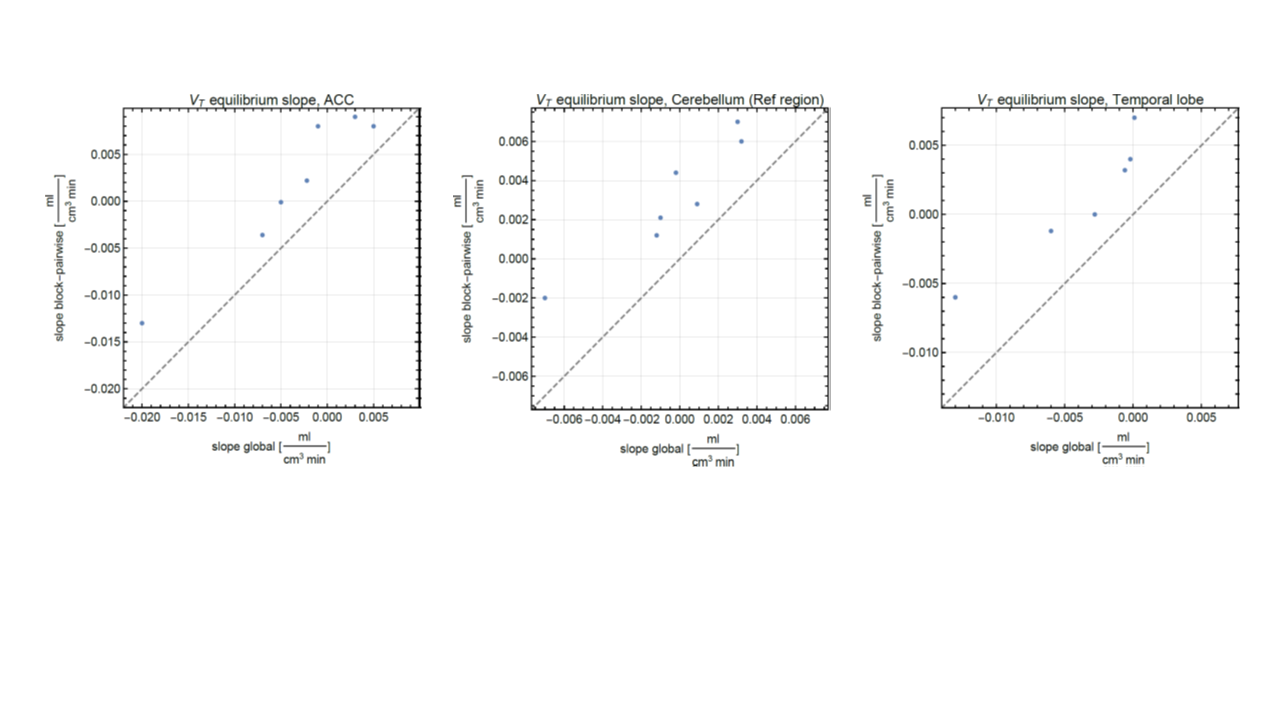

Supplement: S3 Fig — The dashed line represents the identity. (TIF) [file pone.0296357.s003.tif]

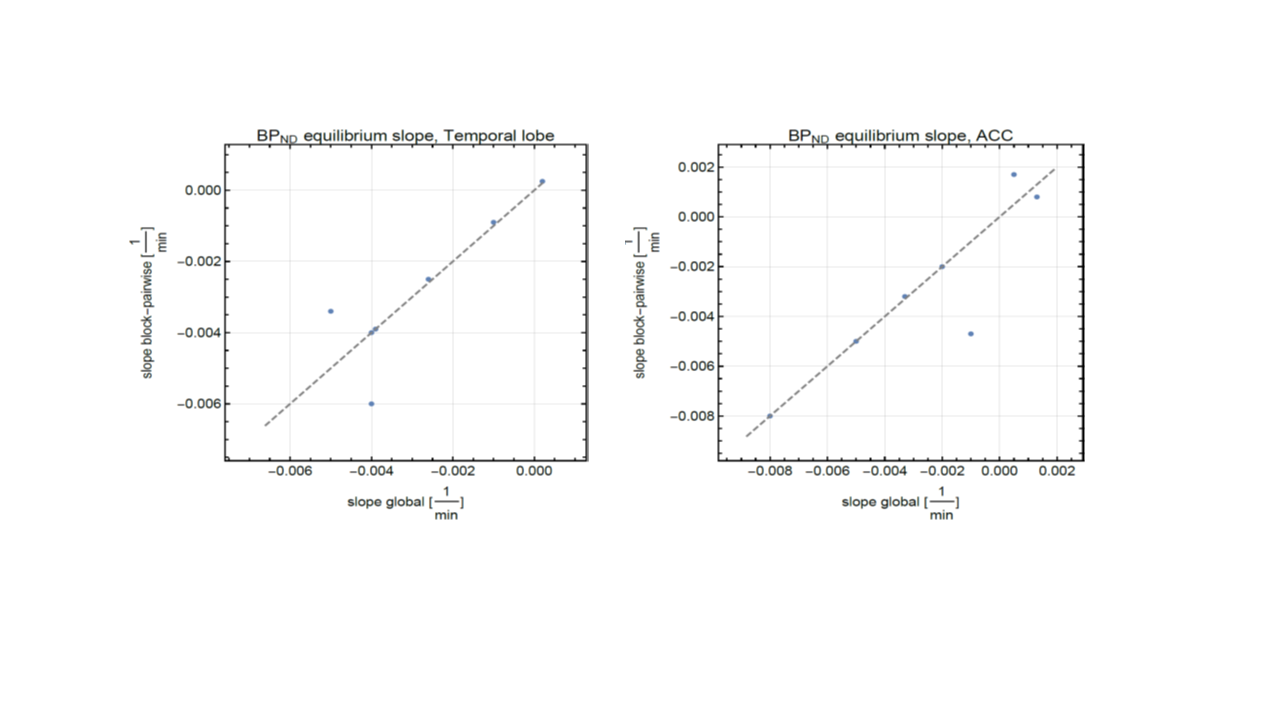

Supplement: S4 Fig — The dashed line represents the identity. (TIF) [file pone.0296357.s004.tif]

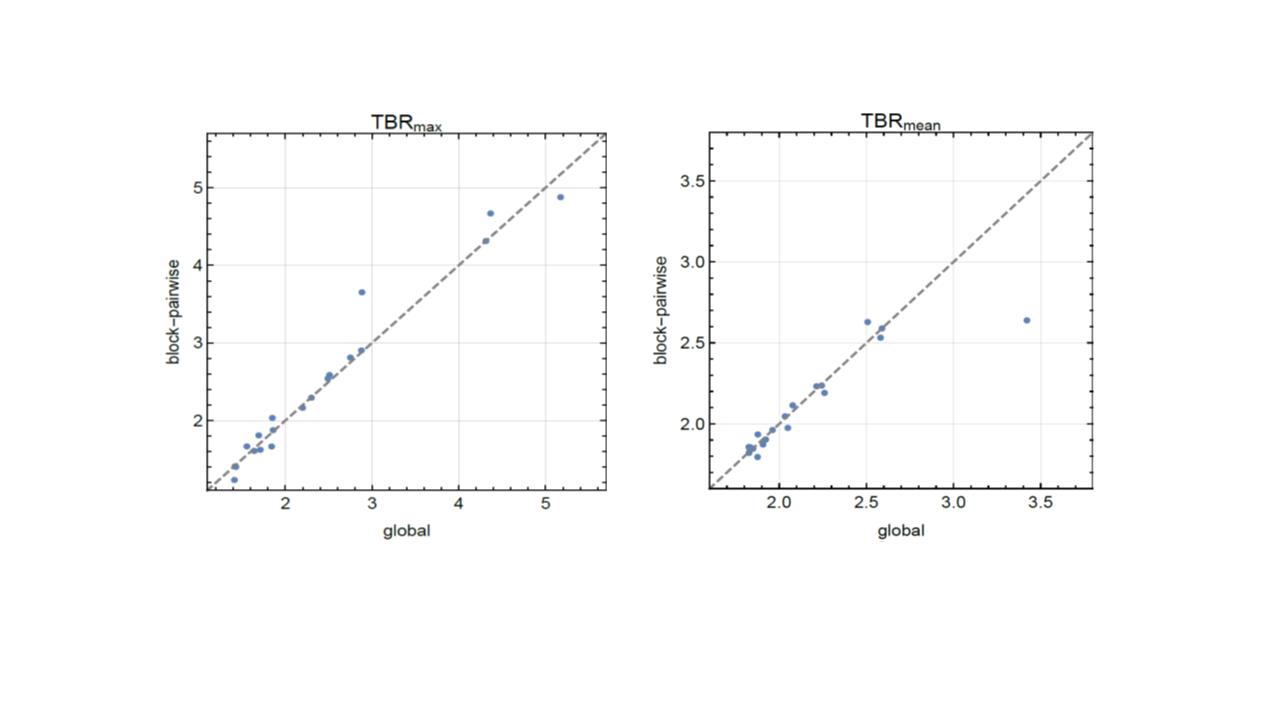

Supplement: S5 Fig — The dashed line represents the identity. (TIF) [file pone.0296357.s005.tif]

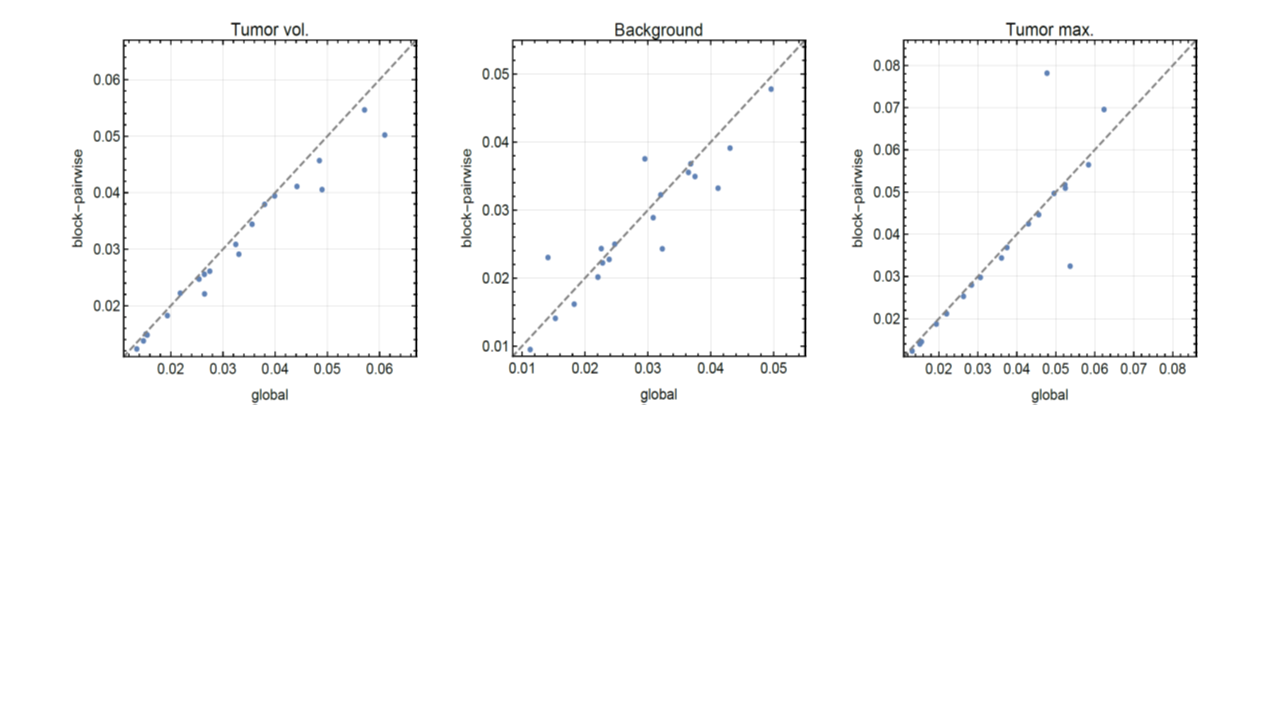

Supplement: S6 Fig — The dashed line represents the identity. (TIF) [file pone.0296357.s006.tif]

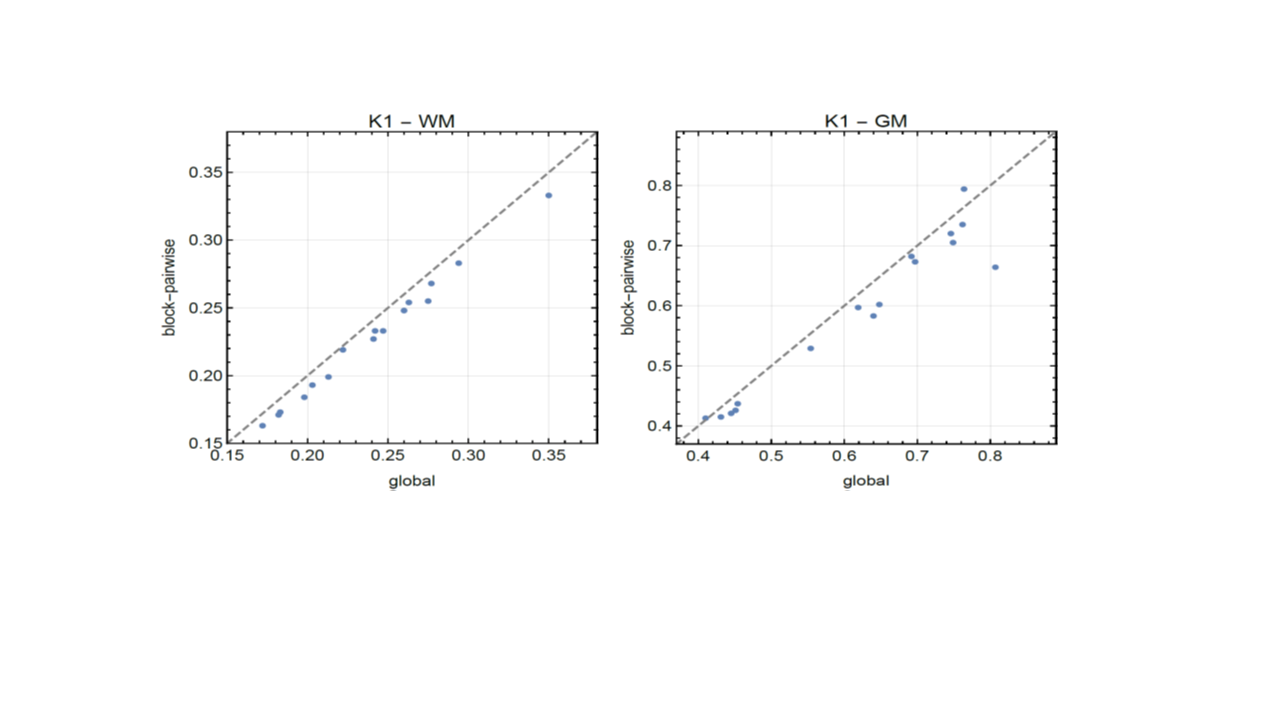

Supplement: S7 Fig — The dashed line represents the identity. (TIF) [file pone.0296357.s007.tif]

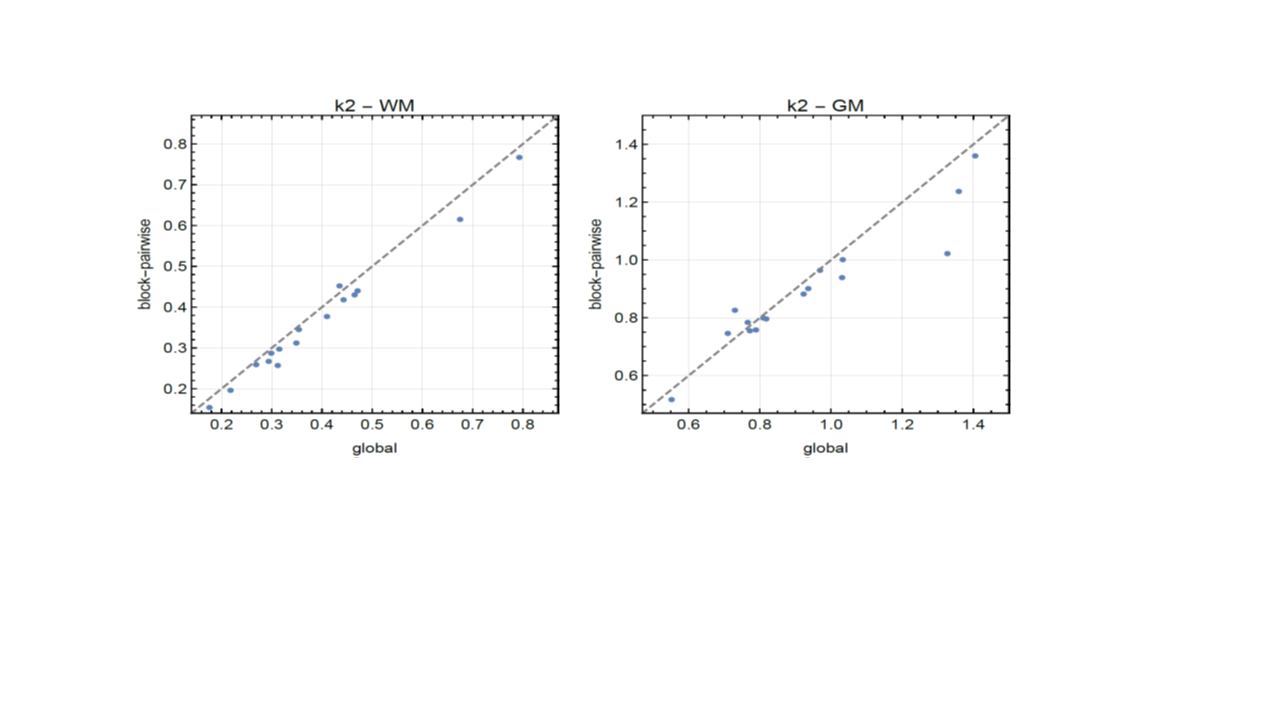

Supplement: S8 Fig — The dashed line represents the identity. (TIF) [file pone.0296357.s008.tif]

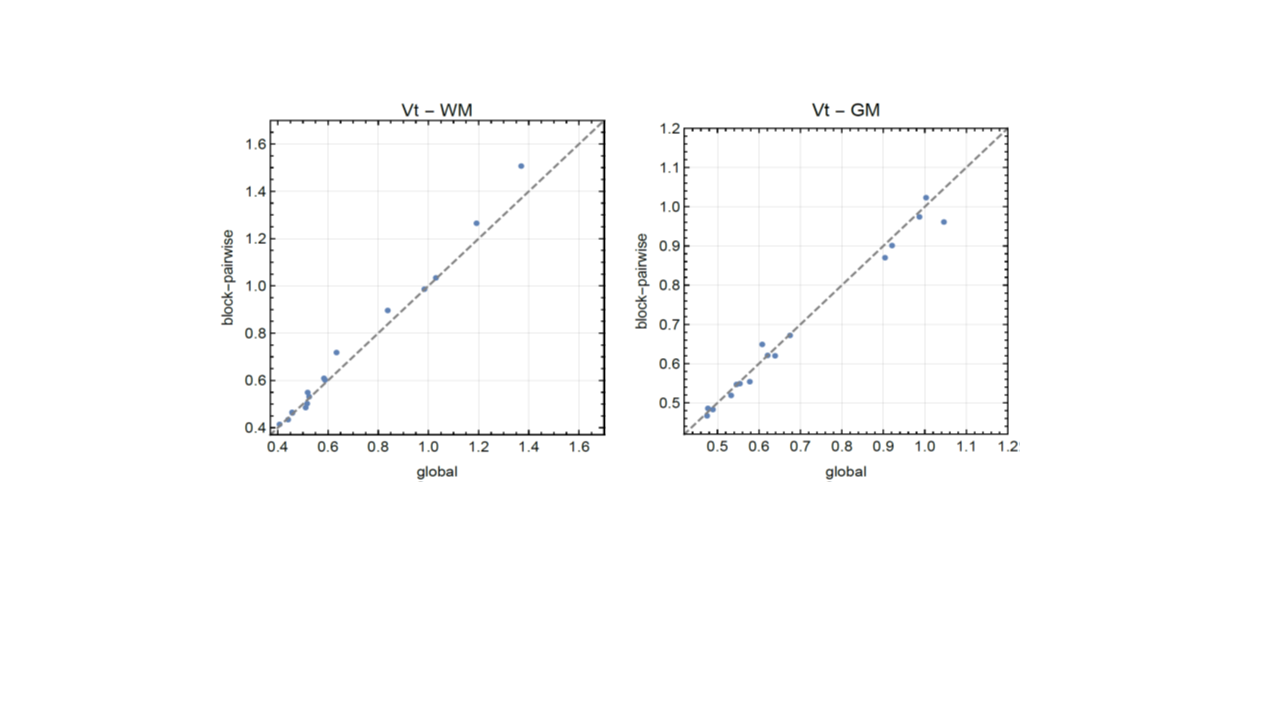

Supplement: S9 Fig — The dashed line represents the identity. (TIF) [file pone.0296357.s009.tif]
